# Protein Palmitoylation Regulates Osteoblast Differentiation through BMP-Induced Osterix Expression

**DOI:** 10.1371/journal.pone.0004135

**Published:** 2009-01-06

**Authors:** Wai Fook Leong, Tielin Zhou, Gek Liang Lim, Baojie Li

**Affiliations:** Cancer and Developmental Biology Division, The Institute of Molecular and Cell Biology, A*STAR (Agency for Science, Technology and Research), Singapore, Singapore; Universität Heidelberg, Germany

## Abstract

Osteoporosis is one of the most common diseases and can be treated by either anti-resorption drugs, anabolic drugs, or both. To search for anabolic drug targets for osteoporosis therapy, it is crucial to understand the biology of bone forming cells, osteoblasts, in terms of their proliferation, differentiation, and function. Here we found that protein palmitoylation participates in signaling pathways that control osterix expression and osteoblast differentiation. Mouse calvarial osteoblasts express most of the 24 palmitoyl transferases, with some being up-regulated during differentiation. Inhibition of protein palmitoylation, with a substrate-analog inhibitor, diminished osteoblast differentiation and mineralization, but not proliferation or survival. The decrease in differentiation capacity is associated with a reduction in osterix, but not Runx2 or Atf4. Inhibition of palmitoyl transferases had little effect in *p53^−/−^* osteoblasts that show accelerated differentiation due to overexpression of osterix, suggesting that osterix, at least partially, mediated the effect of inhibition of palmitoyl transferases on osteoblast differentiation. BMPs are the major driving force of osteoblast differentiation in the differentiation assays. We found that inhibition of palmitoyl transferases also compromised BMP2-induced osteoblast differentiation through down-regulating osterix induction. However, palmitoyl transferases inhibitor did not inhibit Smad1/5/8 activation. Instead, it compromised the activation of p38 MAPK, which are known positive regulators of osterix expression and differentiation. These results indicate that protein palmitoylation plays an important role in BMP-induced MAPK activation, osterix expression, and osteoblast differentiation.

## Introduction

Bone is a dynamic organ and is constantly remodeled. New bones are formed by osteoblasts to replace the old ones, which are resorbed by osteoclasts. A fine balance between bone formation and bone resorption is needed to maintain an optimal bone mass [1], [2]. Indeed, there exist multiple coupling mechanisms between osteoblasts and osteoclasts [3]. For example, osteoblasts can synthesize and secrete cytokines such as M-CSF and RANKL to promote osteoclastogenesis from hematopoietic stem cells of the bone marrow. On the other hand, bone resorption releases TGFβ and BMPs that are trapped in the bone matrix, facilitating osteoblast migration, differentiation and function [4]. Disruption of the balance between bone resorption and formation usually leads to osteosclerosis or osteoporosis [2]. Osteoporosis affects one out of every two women and one out of every four men over age 50, and is regarded as a major public health threat. While there are some anti-resorption drugs in clinical use, such as SERMs and bisphosphonates, there is a lack of anabolic drugs. To date, parathyroid hormone (and teriparatide) and strontium ranelate are the only available anabolic drugs in clinical use [5], [6]. Increasing efforts are being made to search for more efficient anabolic drugs with lesser adverse effects.

Osteoblasts are derived from bone marrow mesenchymal stem cells (MSCs) under the influence of growth factors such as BMPs and Wnts [2], [7]. The two transcription factors that are relatively specific to osteoblast, Runx2 and osterix (Osx), play essential roles in osteoblast differentiation from MSC [8]–[10]. Deletion of either one by gene targeting leads to the loss of mature osteoblasts and lack of calcified bones [11], [12]. On the other hand, ectopic expression of Runx2 or Osx enhances osteoblast differentiation and mineralization [12], [13]. Moreover, there is evidence to support the notion that the levels of Osx determine the differentiation status of osteoblasts [14]–[16]. Given the importance of Osx in osteoblast differentiation and function, it is important to study the regulation of Osx expression. Recent studies show that Osx can be induced by Notch, BMPs, and TNF and its expression is further controlled by posttranslational regulation [17]–[21]. The induction of Osx is believed to mediate the effect of BMPs on osteoblast differentiation. BMPs can transactivate Osx through both the canonical BMP-Smad1/5/8 pathway and the non-canonical BMP-MAPK pathway [17]–[19].

Protein function is affected by its expression level, localization, interaction with other proteins, and its posttranslational modifications. Recent studies indicate that many proteins can be modified by palmitoylation on cysteine residues by a family of proteins that contain a unique *zf*-DHHC domain. At least 24 members have been identified in mammalian genome [22]. This modification regulates protein localization, trafficking, and stability [23]–[25]. To understand the importance of protein palmitoylation in osteoblast biology, we analyzed the expression of the 24 palmitoyl acyltransferases (PATs) by RT-PCR and found some of them were up-regulated when osteoblasts started to differentiate. More importantly, we found that inhibition of protein palmitoylation with a substrate analog inhibitor impeded osteoblast differentiation and function, which are mediated by altered expression of Osx through the p38 MAPK pathway. This study also suggests that palmitoylation modifies proteins downstream of BMPR and upstream of p38 MAPK and this modification might regulate other cellular events that involve this pathway.

## Results

### Expression of palmitoyl transferases during osteoblast differentiation

**Figure 1:**
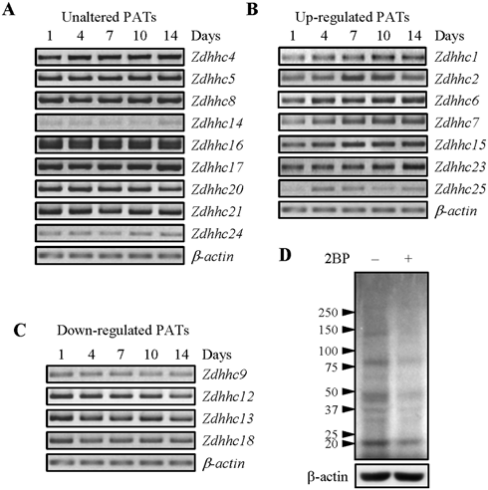
Expression of PATs and protein palmitoylation in osteoblasts. A. RT-PCR analysis of the mRNA levels of the 24 known PATs during osteoblast differentiation. Primary osteoblasts were cultured in the differentiation medium for different periods of time. Total RNA was isolated from these cultures and was then used to carry out RT-PCR. These PATs were classified into unaltered expression (A), increased expression (B), and decreased expression (C), in comparison to the day 1 cultures. D. 2BP was able to inhibit protein palmitoylation in primary osteoblasts. Primary osteoblasts were treated with 100 µM of 2BP for 2 hrs and then ^3^H-palmitic acid was included in the culture medium for 24 hrs. The cells were harvested and the same amounts of total proteins were loaded for control and 2BP treated samples. The SDS-PAGE gel was dried and exposed to x-ray films. The palmitoylated proteins are radio-labeled. Actin was used as a loading control.

Protein palmitoylation plays an important role in regulating localization, trafficking, and degradation of a protein, and its interaction with other proteins. To learn whether protein palmitoylation is involved in osteoblast function, we first analyzed the expression of the 24 potential PATs in calvarial osteoblasts at different stages of differentiation by RT-PCR (Table 1 for primer sequences). It was found that 20 out of 24 PATs can be detected (Fig. 1A–C). More interestingly, DHHC1, 2, 6, 7, 15, 23, 25 were up-regulated in differentiating osteoblasts compared to non-differentiated cells (Fig. 1B), suggesting that these PATs might play an active role in this process. On the other hand, DHHC9, 12, 13, 18 were slightly down-regulated compared to non-differentiated osteoblasts, while DHHC4, 5, 8, 14, 16, 17, 19, 20, and 24 showed no alteration at the mRNA levels (Fig. 1A and 1C). DHHC3, 11, 21, 22 were undetectable, suggesting that their expression in osteoblasts could be very low. Note that DHHC10 does not exist. A recent study showed that in most of the human tissues, 17 of these PATs were expressed [26]. Our previous studies showed that both DHHC19 and DHHC6 were ubiquitously expressed in both mouse and human tissues [27].

**Table 1:** The primer sequences for the 24 mouse PATs and other genes studied here and the sizes of the PCR products.

Gene	Forward	Reverse	Size
Zdhhc1	CACCTGCTCTGCTTCCACAT	CTCAGCTGATGCCGAGTAGT	503
Zdhhc2	GGTCTGCCTGATACTCAAGC	GTTCCATTCCTCCACAGCAC	561
Zdhhc3	TACAAGTGTCCCAAG TGCTG	GGTCCTTCAGACCACATACT	527
Zdhhc4	ACCTTCATCGTCTTGCACCT	GGAGTGAATGTTCTGGTGGA	761
Zdhhc5	ACCCTCACCAGTCCGTTATG	GGTGTAGGTGCAGAGGTGTG	608
Zdhhc6	TTCGGATTGGCTGCGTTTGC	AGTCACCATCACAGGGACAC	612
Zdhhc7	GGTGTGGTTCATCCGAGATG	TTCTCGCTCTTCAGCCTCTC	662
Zdhhc8	CGACAATGGGCTGAAAGCTG	GTGCAGGTAGGGTGAATGGT	666
Zdhhc9	CTACCTCTTCATCCTCTCTC	TCTTCAGGAATGCTGGTGTC	485
Zdhhc11	AGTACTGCCACCTGTGTGAG	TTCGGCGAAAGAGTAGACAC	606
Zdhhc12	GTGCTAAGCTCCCTGCTGCT	GCAGCCTTCTCTCCAGCAAC	665
Zdhhc13	GACTGGCTCTCTGGACTTCA	TAAGCCAAAGCAGCCACACT	531
Zdhhc14	AGCCTGTGTGATAACTGCGT	CATGGTACGGCTATGTGCTA	848
Zdhhc15	CGTTCTCTACTGCCTGTACA	AACCTGCTACGTTTCCGACT	477
Zdhhc16	CTCATCCTCCGAACCTACTC	TCCAGTTGTCCAAGCAGCCA	630
Zdhhc17	ATGGTTCTTCTGGTTCTGGA	TCCTTACACATCCATGGTTG	657
Zdhhc18	TCTCCCTCTCCTTCTTGACG	CTCCTACCATGCTGGCGTCT	431
Zdhhc19	ACCTTCTTCAGTCTCGTCTC	CTACAGTGTTTAGGACGACG	658
Zdhhc20	TCTACACCACATCAGCTTCA	CTGGTTTGCAACAGAAGCTT	589
Zdhhc21	TGCCTGGTTGCCTTAGTGAG	ACAGGATCTTCCAACGAGTG	555
Zdhhc22	GTGACCTTCGTACTGCAGCT	ATTTGTCCTGCTGCTTCGAG	729
Zdhhc23	TGCTGGCACTCTGGTATTAC	CAGCTGGATGAGGAAGATGT	742
Zdhhc24	TTCCTGTGTCTCCTGCTTCA	AGTCACAAGACCCACATCAC	434
Zdhhc25	TCACACCTACGGACTATGCT	CATGGTGCTCACTCACTTTG	576
β-actin	AGATGTGGACAGCAAGCAG	GCGCAAGTTAGGTTTTGTCA	123
Atf4	TTCCACTCCAGAGCATTCCT	CAGGTGGGTCATAAGGTTTG	280
Col1α1	GCAATCGGGATCAGTACGAA	CTTTCACGCCTTTGAAGCCA	484
M-CSF	CTGACACAGGCCATGTGGAG	GAGAGGGTAGTGGTGGATGT	315
Ocn	AAGCAGGAGGGCAATAAGGT	AGCTGCTGTGACATCCATAC	291
Opg	CACCCTGTGTGAAGAGGCCT	GCAGGCTCTCCATCAAGGCA	310
Opn	TCACCATTCGGATGAGTCTG	ACTTGTGGCTCTGATGTTCC	436
Osx	TGAGGAAGAAGCCCATTCAC	ACTTCTTCTCCCGGGTGTG	197
RankL	TACTTTCGAGCGCAGATGGAT	GTACGCTTCCCGATGTTTCAT	483
Runx2	CCGCACGACAACCGCACCAT	CGCTCCGGCCCACAAATCTC	288

### Osteoblasts have many PAT substrates

To confirm that protein palmitoylation does occur in osteoblasts, we labeled OBs with ^3^H-palmitic acid for 24 hours. Total proteins were separated onto SDS-PAGE gels, which were dried and exposed to X-ray films. Osteoblasts clearly showed several radio-labeled bands (proteins with covalent palmitoyl modification). The most prominent ones were at the MW of ∼20, 36, 45, 80, 135 kDa (Fig. 1D). This pattern is different from that of cardiomyocytes, suggesting that different cell types may have specific sets of palmitoylated proteins [28]. Taken together, these results indicate that osteoblasts express many PATs and some proteins are palmitoylated.

### Inhibition of PATs impeded osteoblast differentiation and mineralization, but not proliferation or survival

To study whether protein palmitoylation plays a role in osteoblast function, we intended to inhibit protein palmitoylation with PATs inhibitors and/or to silence PATs with short hairpin RNA. However, too many PATs are expressed in osteoblasts and antibodies against these PATs are mostly unavailable, it was not feasible, at this stage, to silence individual PATs. Instead, we used 2-bromohexadecanoic acid (2-bromopalmitate, 2BP) to block PAT activity. 2BP is a substrate analog inhibitor that should have an effect on most if not all PATs [29]. We found that 100 µM of 2BP was sufficient to repress protein palmitoylation in osteoblast by 80% (Fig. 1D). In the following experiments, we used 2BP at the concentration of 10 to100 µM in osteoblasts to inhibit PAT activities and check the consequences in two aspects: differentiation and mineralization.

**Figure 2:**
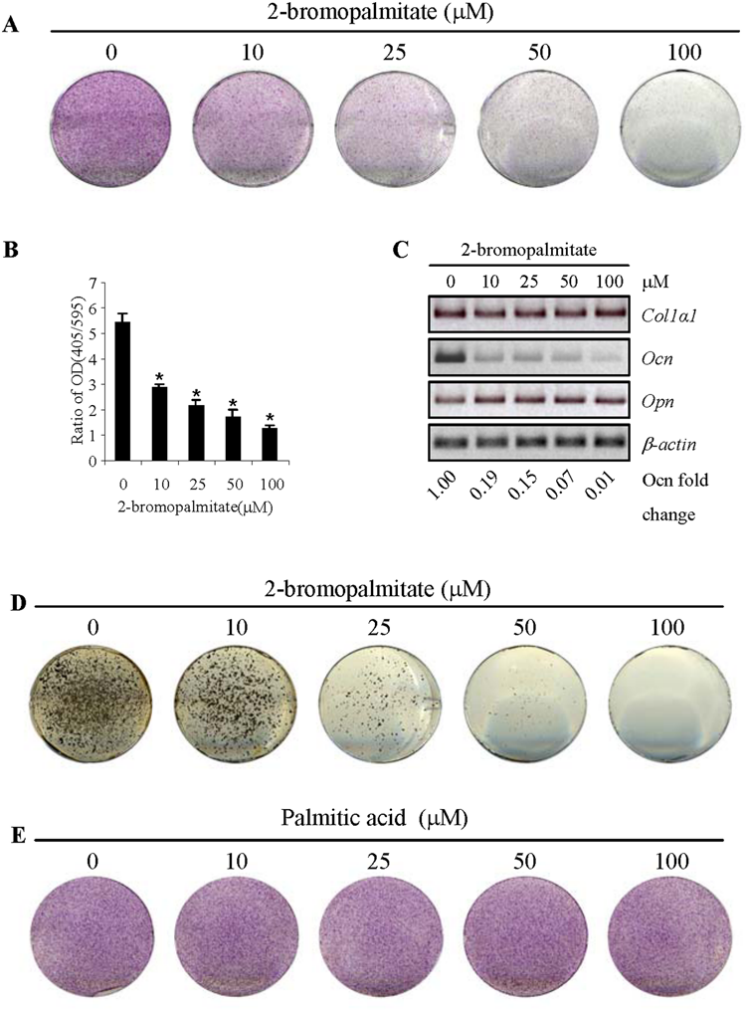
Inhibition of protein palmitoylation impeded osteoblast differentiation. A. The effect of 2BP on ALP staining. Primary osteoblasts were treated with increasing amounts of 2BP for 4 days in differentiation medium and then stained for ALP. B. Quantitation of ALP activities that were normalized to the protein levels of the cells. C. The effect of 2BP on the expression of several osteoblast differentiation markers. The experiments were carried out like Fig. 2A and total RNA were isolated from these cells. RT-PCR was carried out to determine the mRNA levels of these markers. The value of control (lane 1) was set at 1.00. D. The effect of 2BP on bone nodule formation. The experiments were carried out like Fig. 2A. After 21 days in culture, the plates were stained using a Von Kossa method.

We first looked at one of the early markers of osteoblast differentiation, alkaline phosphatase (ALP). It was found that 2BP had a dosage dependent inhibitory effect on ALP expression. Fig. 2A shows the ALP staining of osteoblasts cultured in the differentiation medium, while Fig. 2B shows the quantitative ALP activities that were normalized to the total protein levels. It is obvious that inhibition of protein palmitoylation diminished ALP expression. We then tested the expression of a few other markers by RT-PCR. It was found that 2BP mainly down-regulated the mRNA levels of osteocalcin, but not collagen type 1α or osteopontin (Fig. 2C). More significantly, we found that 2BP severely inhibited bone nodule formation, a later marker of osteoblast differentiation and an indicator of osteoblast bone forming activity (Fig. 2D). These results indicate that protein palmitoylation is required for osteoblast differentiation into mature osteocytes. However, treatment of osteoblast with the same amounts of palmitic acid, the substrate of PATs (>150 µM palmitic acid showed cytotoxicity in primary osteoblasts), showed no significant effect on osteoblast differentiation (Fig. 2E). The reason why higher concentrations of palmitic acid did not enhance osteoblast differentiation could be that the serum provides sufficient amount of palmitic acid for protein palmitoylation.

**Figure 3:**
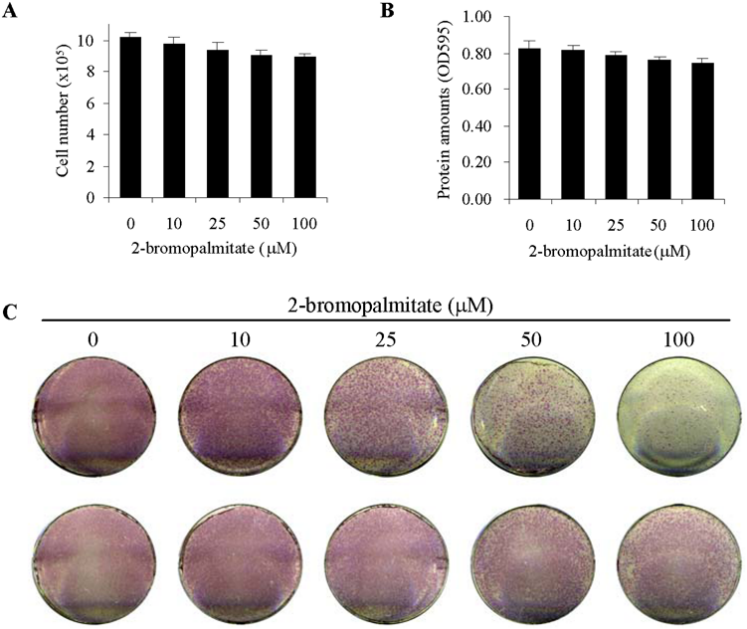
PAT inhibitor show minimal effect on proliferation/survival of osteoblast cultures. A. Primary osteoblast cells were cultured in differentiation medium with different amounts of 2BP for 4 days and the cell numbers in each culture plates were determined by trypan blue exclusion methods. B. The experiments were done like Fig. 3A and the cells were collected and lysed in the same volume of lysis buffer and the protein concentrations were determined by the BioRad method. C. Recovery of 2BP treated cells in ALP expression. Primary osteoblasts were treated with different concentrations of 2BP for three days, washed off, and further cultured in differentiation medium for 4 more days before being stained for ALP (bottom panel), in comparison to the cells that were treated with 2BP all the time (upper panel).

To test whether 2BP has an effect on cell proliferation/survival in the *in vitro* differentiation assays, which may indirectly influence osteoblast differentiation, we first counted the live cells in osteoblast cultures in the presence or absence of 2BP. No significant difference was observed (Fig. 3A). Moreover, 2BP showed no significant effect on the total protein levels of the osteoblast cultures (Fig. 3B). More importantly, removal of 2BP from cell cultures led to a recovery of osteoblast differentiation. Two sets of osteoblast cultures were treated with increasing concentrations of 2BP for three days. One set was continually cultured in the presence of 2BP for 4 more days, while the other set had 2BP washed off and then cultured for 4 more days in the differentiation medium. It was found that the expression of ALP in osteoblasts recovered rather well and this was confirmed by the quantitative ALP assays (Fig. 3C and data not shown). These results indicate that 2BP, at the concentrations used, has little effect on cell proliferation and/or survival, at least for a short term. Instead, it mainly affects osteoblast differentiation.

### Down-regulation of Osx by the PAT inhibitor

**Figure 4:**
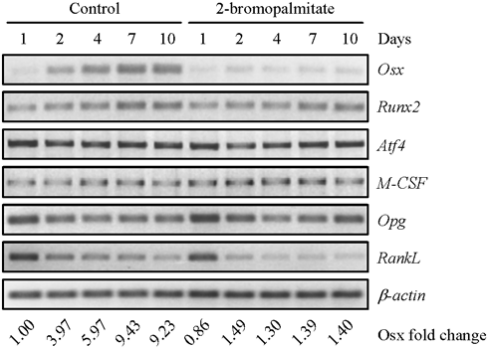
Regulation of Osx by 2BP. Primary osteoblasts were cultured in differentiation medium with or without 100 µM of 2BP for different periods of time. These cells were then collected to isolate total RNA. RT-PCR was carried out to determine the mRNA levels of these proteins. The value of control (lane 1) was set at 1.00.

Osteoblast differentiation is controlled by transcription factors such as Runx2, Osx, and Atf4. Elevation in any of these transcription factors promotes osteoblast differentiation while deletion of any of them leads to defects in osteoblast maturation and bone calcification [11], [12], [30]. We used RT-PCR to monitor the mRNA levels of these transcription factors. Both Osx and Runx2, but not Atf4, showed an up-regulation during osteoblast differentiation, with the increase in Osx mRNA levels being much more pronounced (Fig. 4). 2BP treatment did not significantly affect the mRNA levels of Runx2 or Atf4, suggesting that Runx2 and Atf4 are unlikely to mediate the inhibitory effect of 2BP on osteoblast differentiation. In contrast, 2BP treatment diminished Osx mRNA levels. These results suggest that down-regulation of Osx might mediate the effect of 2BP on osteoblast differentiation.

It is known that osteoblasts express and secrete cytokines that promote osteoclastogenesis [3]. We also tested whether inhibition of protein palmitoylation had any effect on the synthesis of these cytokines. RT-PCR assays show that both OPG and RankL were down-regulated during differentiation while the levels of M-CSF were not altered (Fig. 4). 2BP treatment only slightly decreased the mRNA levels of RankL (Fig. 4). Since during osteoblast differentiation, RankL down-regulation is associated with Osx up-regulation, we believe that the further decrease of RankL induced by 2BP is probably not due to the changes in Osx. These results suggest that inhibition of protein palmitoylation may not greatly affect the ability of osteoblast to promote osteoclastogenesis.

### *p53^−/−^* OBs were resistant to PAT inhibitor in differentiation

**Figure 5:**
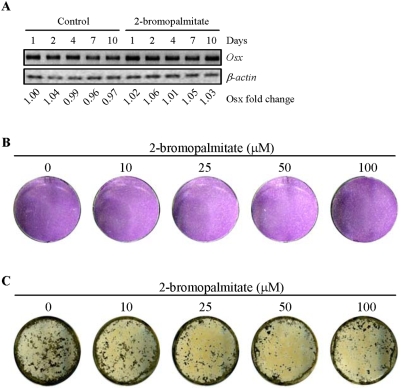
Overexpression of Osx in *p53^−/−^* osteoblasts rendered resistance to the inhibitory effect of 2BP on differentiation. A. 2BP treatment did not significantly down-regulate the mRNA levels of Osx. Primary osteoblasts were cultured in the presence or absence of 2BP for different periods of time and total RNA was isolated and RT-PCR was carried out to determine the mRNA levels of Osx. The value of control (lane 1) was set at 1.00. B. PAT inhibitor did not markedly affect ALP staining in *p53^−/−^* osteoblasts. *p53^−/−^* osteoblasts were treated with different amounts of 2BP for 7 days and then stained for ALP. C. PAT inhibitor did not markedly affect bone nodule formation in *p53^−/−^* osteoblasts. *p53^−/−^* osteoblasts were treated with different amounts of 2BP for 21 days and then stained for mineralization.

To further confirm that Osx mediates the effect of 2BP on osteoblast differentiation, we used *p53^−/−^* osteoblasts that have been shown to have an increased expression of Osx and enhanced differentiation [16], [31], [32]. Moreover, elevated expression of Osx, which was directly repressed by p53, was demonstrated to mediate the effect of p53 on differentiation. We found that the elevated levels of Osx was not further induced during osteoblast differentiation in *p53^−/−^* osteoblast, and that 2BP showed little effect on the mRNA levels of Osx either (Fig. 5A). Consistent with this result, ALP staining was not markedly inhibited by 2BP (Fig. 5B). Moreover, 2BP showed only a slight effect on bone nodule formation in *p53^−/−^* osteoblast cultures (compared to the Fig. 2D) (Fig. 5C). These results indicate that *p53^−/−^* osteoblasts are refractory to the negative effect of 2BP, suggesting that Osx mediated the effect of 2BP on osteoblast differentiation.

### PAT inhibitor compromised BMP2-induced osteoblast differentiation

**Figure 6:**
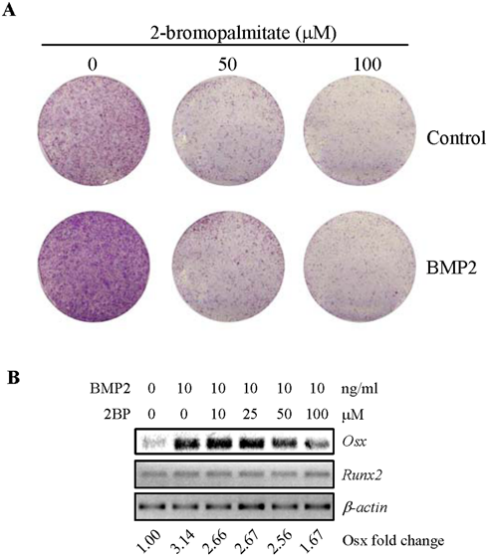
PAT inhibitor decreased BMP2-induced osteoblast differentiation, which was likely mediated by Osx. A. 2BP diminished the ALP expression induced by BMP2. Primary osteoblasts were cultured in the presence or absence of 2BP for 1 day, and then were further cultured in the presence of 50 ng/ml of BMP2 for 4 days. The plates were then stained for ALP. B. 2BP down-regulated BMP2-induced Osx expression. Primary osteoblasts were cultured in the absence or presence of different concentrations of 2BP for a day, and then further cultured in the presence of 50 ng/ml of BMP2 for 16 hrs. The cells were collected and total RNA was isolated, which was used to perform RT-PCR to determine the mRNA levels of Osx and Runx2. The value of control (lane 1) was set at 1.00.

In the *in vitro* differentiation assays, BMPs from the serum or secreted by osteoblasts themselves are the driving force of differentiation. It has been shown that the addition of noggin and chordin to differentiation cultures diminished osteoblast differentiation, which is accompanied by down-regulation of Osx [19]. We then tested whether 2BP has an effect on BMP2 induced differentiation. It was found that 50 ng/ml of BMP2 was able to dramatically up-regulate ALP expression, which was almost abolished by 2BP (Fig. 6A). More interestingly, BMP2 was able to markedly up-regulate the mRNA levels of Osx, which was impeded by 2BP as well (Fig. 6B), supporting the notion that 2BP inhibits osteoblast differentiation by repressing Osx expression. On the other hand, BMP2 had minimal effect on the mRNA levels of Runx2.

### PAT inhibitor negatively regulated the activation of p38 MAPK but not Smad1/5/8

**Figure 7:**
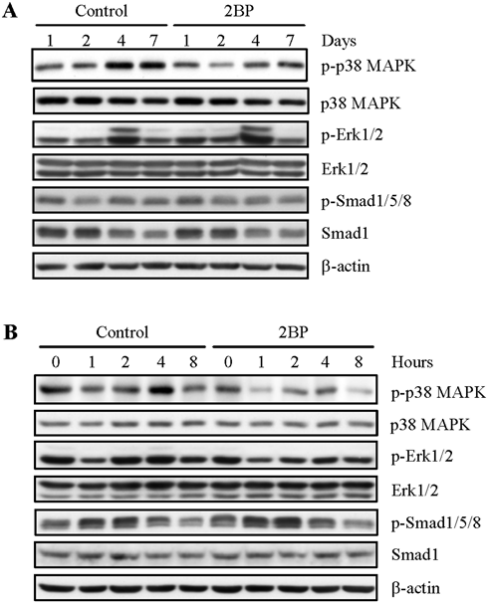
Inhibition of protein palmitoylation compromised p38 MAPK. A. 2BP showed an inhibitory effect on p38 MAPK activation in differentiation assays, without a significant effect on Erk1/2 activation or Smad1/5/8 activation. Primary osteoblasts were cultured in differentiation medium with or without 2BP for different periods of time. Cells were collected for Western blot analysis of the activation of p38, Erk1/2, and Smad1/5/8. B. 2BP also compromised p38 MAPK activation in response to BMP2. Primary osteoblasts were pretreated with PAT inhibitor overnight, and then stimulated with 100 ng/ml of BMP2. Cells were collected at different time points for analysis of p38 and Erk1/2 activation.

How does inhibition of protein palmitoylation regulate Osx expression? Several studies indicate that Osx is under the control of the canonical BMP-Smad pathway as well as the non-canonical BMP-MAPK pathway. We found that 2BP had little effect on Smad1/5/8 phosphorylation in the *in vitro* differentiation assays (Fig. 7A). No significant change was observed in the activation of Erk1/2 either. However, PAT inhibitor seemed to inhibit the activation of p38 MAPK (Fig. 7A). Since BMPs are the driving force of osteoblast differentiation and they activate MAPKs, these results suggest that protein palmitoylation is necessary for the activation of the p38 MAPK pathway, but not the canonical BMP-Smad pathway. This conclusion was supported by the finding that 2BP also compromised BMP2-induced activation of both Erk1/2 and p38 MAPKs (Fig. 7B). The failure of 2BP to affect Erk1/2 activation in differentiation assays (Fig. 7A) suggest that other signaling pathways might have made compensation. The correlation between compromised p38 MAPK activation and the down-regulation of Osx suggests that protein palmitoylation is involved in BMPs-induced p38 MAPK activation, Osx expression and osteoblast differentiation.

## Discussion

This study shows that most of the 24 PATs are expressed in primary osteoblasts, with some showing an up-regulation during differentiation. In addition some proteins are palmitoylated in osteoblasts and the modification can be inhibited by the substrate analog inhibitor, 2BP. It was also found that inhibition of PATs with the inhibitor impeded osteoblast differentiation and function, evidenced by a decrease in the expression of ALP, osteocalcin, Osx, and in bone nodule formation. Moreover, this inhibitor also compromised BMP2-induced osteoblast differentiation. However, treatment of the cells with the same concentration of palmitic acid did not show such effects. In addition, inhibition of PATs showed little effect on osteoblast proliferation and survival under differentiation assay conditions. These results suggest that protein palmitoylation plays an important role in osteoblast differentiation.

How does protein palmitoylation regulate osteoblast differentiation? Our results suggest that Osx might mediate this effect. Firstly, treatment with PAT inhibitor down-regulated Osx expression, not Runx2 or Atf4. Secondly, BMP2-induced osteoblast differentiation was also hindered by the PAT inhibitor and this was accompanied by a decrease in the expression of Osx, but not Runx2. Thirdly, when Osx could not be repressed in *p53^−/−^* osteoblasts, which showed enhanced differentiation due to elevated Osx expression [16], osteoblast differentiation was refractory to the inhibitory effect of the PAT inhibitor. Thus it appears that protein palmitoylation is required for the basal expression of Osx and the induction of Osx by BMP2. These results also support the notion that Osx might be a critical transcription factor that integrates many cues to control osteoblast differentiation. For example, c-Abl, p53, Atm, and p38 MAPK are all involved in osteoblast differentiation and they all regulate the expression of Osx, but not Runx2 [16], [19], [33], [34]. One explanation for the difference between Runx2 and Osx could be that Runx2 induction by BMPs is usually transient and modest, whereas Osx induction can be long lasting and robust (Fig. 4 and 6).

BMPs are synthesized and secreted by osteoblasts and are the main driving force of *in vitro* differentiation [19]. The observation that PAT inhibitor compromised Osx induction by BMP2 suggests that protein palmitoylation might be involved in signaling pathways activated by BMPs. However, PAT inhibitor did not show a marked effect on the canonical BMP-Smad signaling. Instead, it compromised MAPK activation at the basal level and in the presence of BMP2. One possibility is that components of the non-canonical pathway such as Tak1, Tab1/2, Xiap, MAPKKs, are palmitoylated [35]. Another possibility is that BMPRs are palmitoylated and their palmitoylation differentially regulates the Smad pathway and the MAPK pathway. Indeed, we found that BMPRI and II could be palmitoylated. We are currently mapping the palmitoylation sites and studying their role in regulating BMPs triggered Smad and MAPK pathways (Leong et al, unpublished results). Further studies are needed to determine how protein palmitoylation affects MAPK activation through BMP receptors. This is because BMP-Smad signaling and MAPK activation can be complex: i) MAPKs are regulated by many cues including growth factors that are present in the culture medium, and some types of stress; ii) BMPs have been reported to either activate or inhibit MAPKs, depending on the cell type and context [19], [36], [37]; iii) BMPs are known to require cooperation with other signaling pathways to control the expression of target genes due to the nature of Smad binding elements (only 4 bp) [38]; iv) a crosstalk exists between BMP-Smad signaling and MAPK [38], [39]. Thus it is important that caution be exercised in interpreting these results. Nevertheless, this study provides a new dimension in p38 MAPK activation and osteoblast differentiation. Future study will focus on identifying the palmitoylation target and studying the function of the palmitoylation in regulating Osx expression and osteoblast differentiation.

In summary, this is the first study to show that protein palmitoylation plays an important role in osteoblast differentiation and function. It seems that the potential palmitoylation substrates might regulate the MAPK pathway, especially p38 MAPK, to regulate Osx expression and osteoblast differentiation. Given the importance of BMPs activated signaling pathways in bone remodeling and cancer development, it is worth the effort to further study how protein palmitoylation affects these signaling events.

## Materials and Methods

### Isolation, culture and treatment of calvarial osteoblasts

To prepare primary osteoblasts, calvaria were excised from 4–5 newborn pups (*p53^−/−^* and wild type on a 129/Sv background), washed in PBS and digested in MEM alpha medium containing 0.1% collagenase type V & 0.05% trypsin-EDTA for 15 min at 37^o^C five times. The supernatant from the first digestion was discarded and supernatants from the last four digestions were pooled. The cells were washed and plated onto 6 well plates and grown in MEM alpha medium supplemented with 15% FCS (Research Sera) and glutamine until confluent. The osteoblast cultures were amplified to passage 3 before use in further experiments. The mice were kept at the Biological Resources Center (BRC) of Singapore and were used for the experiments following protocols approved by the Institute of Molecular and Cell Biology and BRC. A palmitate analog inhibitor, 2-bromohexadecanoic acid (Merck, Darmstadt, Germany) was dissolved in 100% ethanol at a stock concentration of 100 mM and added to cells at the respective concentrations with ethanol as the control.

### Alkaline phosphatase (ALP) staining and quantitation

Calvarial cells were seeded on 12-well plate at a density of 1.5×10^5^ cells in differentiation medium (growth medium supplemented with 50 µg/ml ascorbic acid and 10 mM β-glycerophosphate) and incubated at 37°C for 4 or 7 days. For ALP staining, the differentiation medium was removed and the cells were fixed with fixative solution [60% v/v acetone and 0.8% v/v citrate concentrate (Sigma-Aldrich, St. Louis, MO)] for 30 seconds before rinsing with deionized water for 45 seconds. For the biochemical assay, cell layer was rinsed twice with Tris buffered saline [20 mM Tris in 0.9% NaCl, pH 7.4] before harvesting in 500 µl of 50 mM Tris, pH 7.4 buffer. The cell suspension was sonicated for 20 seconds. The ALP activity was assayed in 221 assay buffer [0.1 M 221 buffer (Sigma-Aldrich), 10 mM MgCl_2_, pH 10.3] with 10 mM p-nitrophenylphosphate (Sigma-Aldrich) as the substrate. The reaction was stopped upon the addition of 0.3 N NaOH and the absorbance was read at 405 nm. The enzyme activity was then normalized with the protein concentration.

### Bone nodule formation

Calvarial cells were seeded as for ALP staining but were incubated for 14 and 21 days. Cells were rinsed twice with Tris buffered saline and followed by fixing with 4% formalin for 5 to 10 minutes. The fixed cells were then rinsed twice with ddH_2_O. Mineral deposition was stained with 5% silver nitrate and exposed under UV until desired intensity was achieved before washing with ddH_2_O.

### RNA isolation and Quantitative RT-PCR

Total RNA was extracted from cells after rinsing twice with ice-cold PBS and purified with TRIzol® reagent (Invitrogen) according to manufacturer's protocol. cDNAs were synthesized from 1 µg of total RNA using Reverse-It™ RTase Blend (Abgene, Epsom, UK) with Oligo(dT)_15_. The detection and quantification of target mRNA was performed with semiquantitative RT-PCR. The primer sequences for the respective genes are shown in Table 1. The amplification for each mRNA was performed in the linear range for RT-PCR by optimizing the template concentration and limiting the amplification cycles to below 30 to ensure exponential amplification.

### Western blot analysis

The cells were rinsed twice with ice-cold PBS and harvested by scraping in 80 µl RIPA buffer [50 mM Tris, pH 8.0, 150 mM NaCl, 1 mM EDTA, 1% (v/v) NP-40, 0.5% (w/v) NaDOC, 0.1% SDS, 1 µg/ml aprotinin, 1 µg/ml leupeptin, 1 µg/ml pepstatin, 2 mM PMSF, 2 mM NaF, 4 mM Na_3_VO_4_], followed by rocking at 4°C for 30 minutes. The cell lysates were then clarified by centrifugation at 12,000 g for 10 minutes. The proteins were quantified using DC protein assay (Bio-Rad, Hercules, CA). 60 µg proteins were resolved by SDS-PAGE and then electrophoretically transferred to PVDF membrane. The membrane was blocked with 5% non-fat milk for an hour and probed with the respective primary antibodies overnight at 4°C. After incubation, the membrane was washed thrice with TBST for 10 minutes each and probed with respective secondary antibodies for an hour. Finally, the membrane was washed thrice with TBST and target proteins were detected using ECL kit (GE Healthcare, Buckinghamshire, UK). The primary antibodies used for analysis were anti-Erk1/2, phospho-Erk1/2, p38 MAPK, phospho-p38 MAPK, p53, phospho-Smad1/5/8 (Cell Signaling, Danvers, MA), anti-Smad1 (Millipore, Billerica, MA), anti-β-actin (Sigma-Aldrich).

### Protein labeling with ^3^[H]-palmitic acid

Calvarial cells were pretreated with 100 µM 2-BP for 4 hours before the treatment with 50 µCi [9, 10-^3^H(N)]-palmitic acid (Perkin Elmer, Waltham, MA) for 24 hours. Total proteins were harvested, quantitated and resolved by SDS-PAGE. The gel was then fixed with acetic acid and methanol for an hour. This was followed by impregnating the gel with EN^3^HANCE™ (Perkin Elmer) for an hour and subsequently, precipitating the scintillators with cold water containing 10% polyethylene glycol 6000 for an hour. The treated gel was sandwiched between two layers of cellophane sheets and air-dried before exposing to X-ray film for 3 weeks.

### Quantitation and statistical analysis

RT-PCR (negative images of gels) was scanned with a Molecular Dynamics scanning densitometer. The relative levels of mRNA of interest were then determined by measuring the intensity of the corresponding bands. All values were averages of three experiments and were normalized to the constitutive expression of the housekeeping genes β-actin. Statistical analysis was performed using an unpaired *t* test (STATISTICA software; StatSoft, Inc.). Significant association was defined when * P<0.05 compared with control.
